# A Clinical Retrospective Study of Recurrent Painful Ophthalmoplegic Neuropathy in Adults

**DOI:** 10.1155/2021/9213852

**Published:** 2021-12-17

**Authors:** Cheng Li, Xinyi Huang, Xiao Tan, Yannan Fang, Jianhua Yan

**Affiliations:** ^1^State Key Laboratory of Ophthalmology, Zhongshan Ophthalmic Center, Sun Yat-sen University, Guangzhou 510060, China; ^2^Intensive Care Unit of the Seventh Affiliated Hospital, Sun Yat-sen University, Shenzhen 518000, China; ^3^Shenzhen Aier Eye Hospital Affiliated to Jinan University, Shenzhen 518000, China; ^4^Guangdong Key Laboratory for Diagnosis and Treatment of Major Neurological Diseases, Department of Neurology, National Key Clinical Department and Key Discipline of Neurology, The First Affiliated Hospital, Sun Yat-sen University, Guangzhou 510060, China

## Abstract

**Introduction:**

Recurrent painful ophthalmoplegic neuropathy (RPON) is quite rare and usually occurs in children. In this report, we describe the clinical features, diagnosis, and treatment of RPON in adults.

**Methods:**

A retrospective review was conducted of all RPON cases seen and treated at the Zhongshan Ophthalmic Center of Sun Yat-sen University and the Department of Neurology of the First Affiliated Hospital, Sun Yat-sen University, Guangzhou, China, over the period from January 2016 to May 2020.

**Results:**

A total of 8 patients (3 males and 5 females) with a mean age of 42.9 years (range: 23–64 years) met the diagnostic criteria of RPON. Headaches were present prior to the onset of ophthalmoplegic neuropathy in 50% of these patients, while in the remaining 50%, headaches occurred simultaneously with eye symptoms. The degree of these headaches was described as being mild or moderate. Abnormalities involving cranial nerve III were the most frequently reported pathologies (6 cases, 75%), followed by nerve VI (4 cases, 50%) and then nerve IV (1 case, 12.5%) (more than one nerve was affected in some cases). Following either with glucocorticoid treatment or with observation only, symptoms and signs within all 8 patients completely dissipated within 3–28 days.

**Conclusions:**

All adult cases of RPON along with their clinical features as reported here were similar to those of children.

## 1. Introduction

Painful ophthalmoplegia (PO) is a well-known entity, presenting with orbital pain combined with any combination of ipsilateral ocular motor palsies, oculosympathetic paralysis or sensory loss in the distribution of the ophthalmic, and occasionally, maxillary division of the trigeminal nerve [1, 2]. However, PO is diffusely defined with a varied etiology, most often involving trauma, neoplasm, and vascular and/or inflammatory disorders [3]. Recurrent painful ophthalmoplegic neuropathy (RPON) represents a type of PO but is distinguished by repeated attacks and an absence of any known etiology, although nerve enhancement or demyelinating cranial neuropathy has been suspected in some cases [4–9]. Clinically, the diagnosis of RPON is challenging due to its rarity (annual incidence of 0.7 per million [10]), indistinct pathological mechanisms, and an occurrence which is predominantly confined to children [11]. As a result, ophthalmologists often ignore RPON in clinical practice and thus seldom treat this condition in a proper and timely manner. Therefore, in this report, we conducted a retrospective review of RPON cases at our ophthalmic and neurology centers to compile a characterization of the clinical features, diagnosis, and treatment of RPON. This review was limited to RPON cases in adults as this group has been largely ignored with regard to this condition.

## 2. Materials and Methods

The Ethics Committee of the Zhongshan Ophthalmic Center, Sun Yat-sen University, approved this retrospective study, which was conducted according to the principles expressed in the Declaration of Helsinki. The committee specifically waived the need for consent. Patients' consent was obtained before medical information was collected.

Inclusion criteria: this retrospective review consisted of all adult RPON cases who were seen and treated over the period from January 2016 to May 2020 at either the Zhongshan Ophthalmic Center of Sun Yat-sen University or the Department of Neurology of the First Affiliated Hospital, Sun Yat-sen University, Guangzhou, China. The diagnostic criteria for inclusion within the study included (1) acquired limitations in ocular motility, strabismus, and diplopia, often with the involvement of cranial nerves III, IV, and VI, (2) ipsilateral headache, either before/after or simultaneously with the strabismus, (3) all negative systemic and local etiological assessments, such as blood biochemistry, metabolic tests, and higher MRI orbital and brain imaging, and (4) repeated attacks of ocular motility restrictions and headache. Exclusion criteria: All PO patients with clear etiologies of eye movement disorders and only one attack of those as described above were excluded from the study.

### 2.1. Examination, Surgery Method, and Systemic Glucocorticoids Administering

The following data were collected: medical history, duration of diplopia and strabismus, headache, response to steroids, response to strabismus surgery, and follow-up times. Ophthalmic examinations included the best corrected visual acuity (BCVA), intraocular pressure (IOP), slit-lamp microscope examination, retinoscope examination, and assessment of the abnormal head position. In the evaluation of ocular motility, duction was graded on a standard four-point scale from −4 (underaction) to +4 (overaction) with 0 being normal. In patients receiving surgical correction, their deviation angles were assessed by the prism and alternate cover test (PACT) at distance (6 m) and at near (33 cm). Stereoacuity was measured with the use of the Randot test at distance (5 m) and by the Titmus test at near (33 cm). Surgical details included the muscle receiving surgery, type of surgery performed, and amount of recession and/or resection. A full investigation of each patient consisted of a general medical examination, orbital and brain imaging using higher magnetic resonance imaging (MRI) (including contrast-enhanced cerebral MRI and MRA), blood biochemistry and metabolic tests, functional assessments of the liver, kidney, and thyroid, autoimmune-related factor tests (especially for myasthenia gravis), inflammation-related tests, chest X-ray, cardiovascular disease risk examination, and infection via IgM screening.

When systemic glucocorticoids were administered in our cases, the initial doses were 80–120 mg/day of methylprednisolone by IV drip for 3 days, followed by a change to oral prednisone at 40–60 mg/day for one week. If headaches and cranial nerve palsy were completely resolved, the drug therapy was terminated, while in patients showing partial resolutions, the oral prednisone was gradually reduced by a dose of 10 mg per week. In addition, each patient received supplemental treatments consisting of neurotrophic drugs, such as cattle encephalon glycoside, as well as ignotin, vitamin B1, B6, and mecobalamin.

In patients with obvious residual cranial nerve palsy after repeated attacks, strabismus surgery was employed after the deviation was stable for more than 6 months. The surgical methods used were similar to those for routine paralytic strabismus.

## 3. Results

A total of 8 patients were recruited in this study. There were 3 males and 5 females with a mean age of 42.9 years (range: 23–64 years). The left eye was affected in 4 cases, the right in 3 cases, and both eyes in 1 case. The mean time of RPON duration was 8.25 years (range: 2–22 years). The main symptoms in all cases consisted of headaches, diplopia, and ocular motility restrictions. The initial frequency of RPON attacks ranged from 0.5–6 times/year. Frequencies of these attacks did not change in 4 of the cases, increased in 3 cases and decreased in 1 case. Headaches occurred prior to the onset of ophthalmoplegic neuropathy in 50%, while in the remaining 50%, the headaches occurred simultaneously with eye symptoms. Two patients (cases 1 and 4) presented with bilateral headaches and both cranial nerves III and VI were affected in these 2 cases. In Case 4, the affected cranial nerve changed from III to IV to VI as a function of differing attacks. Headaches were ipsilateral to the involved cranial nerve in all but one case. This patient (case 3) showed right cranial nerve III neuropathy with left head pain, accompanied by tactile hypersensitivity on the left side of the face. Two patients reported that their headaches and cranial nerve palsy often occurred after a cold or when feeling fatigued. Headaches located at the brow arch and forehead ipsilateral to cranial nerve palsy were experienced by two patients and six cases presented with ipsilateral migraine. All patients reported that the degree of their headaches was mild to moderate and was tolerable. Cranial nerve III was the most commonly affected nerve (6 cases, 75%), followed by nerve VI (4 cases, 50%) and nerve IV (1 case, 12.5%). In some cases, more than one cranial nerve was affected with 1 case having perturbations in cranial nerves III and VI (case 1), while cranial nerves III, IV, and VI were all involved in one case (case 4). In addition, we found that one patient (case 3) showed an involvement of the trigeminal nerve (first branch). Brain and orbital MRI examinations of all patients showed no obvious structural abnormalities. However, we did observe that two patients showed arterial contact with the oculomotor nerve. Nausea and vomiting were experienced in 2 cases and photophobia in 4 cases.

Five patients received systemic glucocorticoid therapy accompanied with nutritional nerve drugs. Among these 5 patients, one (case 1) had previously received glucocorticoid therapy which resulted in improvements in her headaches. In another case (case 6), strabismus surgery with 5 mm superior rectus recession in the left eye was performed due to a long-existent hypertropia of the affected left eye, which was unresponsive to repeated administrations of systemic glucocorticoids. The MRI did not show any nerve atrophy in this case. In three patients, who did not receive any drugs or treatments, all symptoms and signs dissipated after 3–28 days.

For all 8 patients, either with glucocorticoid treatment or with observation only, all symptoms and signs completely dissipated after 3–28 days; however, residual strabismus remained in 3 patients. Follow-up times ranged from 1.0–3.8 years (average: 2.23 years). All demographic data, initial attack frequencies, frequency changes, headaches, cranial nerves involved, treatments, and final outcomes are summarized in Table 1.

## 4. Discussion

RPON is a rare neurological disorder characterized by recurrent bouts of headaches and ophthalmoplegia. Current descriptions of this condition are mostly published in the form of case reports. The mean age of cases in our series was 42.9 years (range: 23–64 years). As we focused on adult cases, this age was substantially older than that of the mean of 8 years of age from a previous review [12]. However, our female to male ratio of 5 to 3 as well as the site of involvement being evenly distributed between the right and left sides was similar to the results of that review [12]. In general, the clinical signs of RPON manifest as episodes of ipsilateral headache followed by palsy of one or more ocular cranial nerves that begins immediately or up to 14 days after the onset of the headache, depending upon the cranial nerve affected. Ophthalmoplegia may persist for weeks to months and is typically reversible [12, 13]. Most patients recover completely within days to weeks, but in a small number of these cases, patients are left with persistent neurological deficits [14]. Cranial nerve III is the most commonly affected and associated with cases of mydriasis and ptosis. The next most frequently perturbed cranial nerve is VI, which only manifests as esotropia and abduction deficits, while the involvement of cranial nerve IV is rare [15]. In our cases, the incidence of cranial nerve III involvement was 75.0%, cranial nerve VI 50.0%, and cranial nerve IV 12.5%, which is consistent with reports in the literature [15]. In one unique case (case 4), the cranial nerves involved changed from the nerve III to IV to VI as associated with different episodes of attack. In addition, we also observed a patient with trigeminal involvement (first branch). Taken together, these findings suggest that the pathogenic factors of RPON may involve a relatively wide range of cranial nerves.

For the cases included in this report, the diagnosis of RPON was made after ruling out all other possible conditions which may result in headache and cranial nerve palsy. As based on the description contained in the International Classification of Headache Disorders (3rd Edition), the diagnostic criteria of RPON require that patients must have at least two attacks characterized by unilateral headache ipsilateral to the paresis of one or more of three ocular cranial nerves (III, IV, or VI), and that other orbital, parasellar, or posterior fossa lesions have been excluded as verified by appropriate investigations [16]. With the exception of case 3 who experienced right cranial nerve III neuropathy and left head pain, for all other cases in our report the headache was ipsilateral to the involved cranial nerve. The older terminology of ophthalmoplegic migraine seems outdated as the headaches are not always fully migrainous in character which might then argue against a migrainous cause [17]. The existing guidelines suggest that RPON is a painful cranial neuropathy rather than migraine, however, the available evidence does not justify such a simple view as some cases do suggest a migrainous component in this rare condition [17]. Six of 8 cases in our series presented with ipsilateral migraine. Therefore, RPON was sometimes considered as a migraine variant by some authors. The basis for this conjecture is that one or more ocular cranial nerve palsies, most often cranial nerve III, can be associated with a migrainous headache [18]. In our patients, no clear pathogenesis could be defined, however, it is worth noting that two patients had arterial contact with the oculomotor nerve. The clinical significance of this contact and whether it may be a potential cause remain unclear.

A confirmed diagnosis of RPON necessitates that all other organic changes be excluded, in particular, Tolosa-Hunt syndrome (THS) [7, 8]. THS is described as unilateral orbital or periorbital pain associated with the paresis of one or more of the III, IV, and/or VI cranial nerves and results from granulomatous inflammation in the cavernous sinus or the superior orbital fissure/orbit [16]. Like that for RPON, systemic administrations of corticosteroids are very effective in treating THS. Overall, the diagnostic criteria of THS are more focused on granulomatous inflammations within the sites described above and as demonstrated by MRI or biopsy [17], while the emphasis of RPON is on undetectable causes and extemporaneous relief of the attacks. We feel that ROPN is underdiagnosed and the incidence of this condition will substantially increase when clinicians, especially ophthalmologists, become more familiar with its clinical features and characteristics.

The exact pathogenesis of RPON has yet to be fully elucidated, and only with a more complete understanding of its mechanisms, it will be possible to better initiate effective treatments. Some possible etiologies may include autoimmune hypothyroidism and schwannoma [19–21], nerve compressive conditions (ethmoidal mucocele, neurofibromatosis type 2, recurrent hemorrhage in pituitary adenoma, pseudotumor cerebri, carotid basilar anastomosis, and a Reye-like syndrome), essential mixed cryoglobulinemia, and postvaccinal [22]. Smith and Schuster suggested that a relapsing-remitting inflammatory or demyelinating process and permanent nerve damage may accumulate with repeated attacks [18]. Based on our experience, we proposed that the repeated attacks of a microscopic origin of vascular microangiopathy, especially in patients with diabetes mellitus, hypertension, and atherosclerosis, may be an important source for this condition. It is clear that the identification of the etiology of RPON is sorely needed for a correct diagnosis and effective treatment of this condition.

Our cases were unusual as all the involved cases had an onset of RPON in adulthood, even if the earliest onset time in history is inquired in detail. The mean time duration of RPON in these adults was 8.25 years (range: 2–22 years), and the frequency of attacks ranged from 0.5 to 6 times per year. This frequency remained constant in 4 cases, increased in 3 cases, and decreased in 1 case. To our knowledge, there is no other report of decreased frequencies of attacks in RPON. More commonly, RPON is a disease prevalent in children and can persist into adulthood [12, 18, 23–25]. The median age at the time for the first ophthalmoplegic migraine attack was 8 years, and the age at presentation as indicated in the most recent report on RPON ranged from 1 to 74 years [12]. As RPON is more prevalent in children, the adult cases investigated in this report may be somewhat biased.

Although the patients in our report improved to varying degrees after receiving systemic glucocorticoid therapy, patients with RPON often do not show any evidence of a systemic inflammatory process and can spontaneously recover without any treatment. Therefore, the issue of whether or not hormone therapy should be used remains controversial. There is not an exact use/dose regimen established for the glucocorticoid treatment of RPON. As these treatments and evaluations of RPON cases have not been conducted in blinded investigations in any of the previous literature, further clinical investigations will be required to evaluate the efficacy and safety of glucocorticoid therapy in the future. Unfortunately, these investigations are hampered by the limited pool of RPON cases available for conducting such studies.

There are some limitations in our study, notably the small sample size of subjects and the retrospective nature of this review. The follow-up time periods of our patients were relatively short, and there were variations in the regimens of systemic glucocorticoid treatments among different clinicians. In addition, it was not possible to compare final outcomes between groups treated or not with systemic glucocorticoid.

In summary, RPON is a rare, complex disorder with possible features of inflammatory neuropathy and an unclear association with migraines. Some patients with oculomotor nerve palsy, in the absence of a clear etiology, may be experiencing RPON. The findings that the headaches experienced in our patients were not particularly severe may result in both clinicians and patients ignoring the significance of this potentially important symptom. Therefore, it is important to attend to reports of mild or slight headaches. In addition, a previous history of palsy in the same or other cranial nerves needs to be recorded. Interestingly, some patients can recover spontaneously without any treatment, while others only show improvements with glucocorticoid therapy. However, after repeated attacks, the degree of strabismus will be constant, and surgery may eventually be required for some of these patients. Additional case reviews and further research directed toward understanding the underlying pathogenesis of this unusual condition in adults are warranted.

## Figures and Tables

**Table 1:** The clinical findings and final outcomes of RPON patients.

Case	Age (year)/gender/eye	Time of duration	Initial frequency	Frequency changes	Headache	Cranial nerve	Treatment	Final outcome	Follow-up (year)
1	54/M/L	5 years	2 times/year	No change	Headache appeared 2 to 3 days before strabismus, indescribable pain in the entire head	III, VI	Glucocorticoids, neurotrophic drugs	Symptom completely disappeared	3
2	27/F/R	2 years	1 time/year	No change	Headache and strabismus occurred simultaneously	VI	Glucocorticoids, neurotrophic drugs	Symptom completely disappeared	3
3	33/M/R	3 years	4 times/year	No change	Mild left head pain, accompanied by tactile hypersensitivity on the left face, headache and strabismus occurred simultaneously	III, V	Glucocorticoids, neurotrophic drugs	Residual strabismus	3.8
4	62/F/B	10 years	3 times/year	No change	Whole head pain, moderate and tolerable, occured at the same time with strabismus	III, VI, IV	Glucocorticoids, neurotrophic drugs	Residual strabismus	3
5	39/F/L	22 years	0.5 time/year	2 times/year in recent 5 years	After catching a cold, there was a tolerable headache at the left brow arch and forehead, and squint appeared 1 day later	III	Observation only	Residual strabismus	1
6	41/F/L	5 years	6 times/year	9 times/year in recent 2 years	Tolerable headache on the left side occurred at the same time with strabismus	III	Glucocorticoids, strabismus surgery	Symptom completely disappeared	2
7	64/F/R	3 years	6 times/year	24 times/year in recent 1 year	Moderate headache on the right side first, and then squint appeared 2 days later	VI	Observation only	Symptom completely disappeared	1
8	23/M/L	16 years	6 times/year	1 time/3 years in recent 4 years	Moderate headache on the left forehead after catching a cold or fatigue, then squint 1 to 2 days later	III	Observation only	Symptom completely disappeared	1

M = male; F = female; R = right eye; L = left eye; B = both eyes.

## Data Availability

All the data generated or analyzed during this study are available in the medical record system of Zhongshan Ophthalmic Center and the First Affiliated Hospital, Sun Yat-sen University.
